# Postoperative standard chemoradiotherapy benefits primary glioblastoma patients of all ages

**DOI:** 10.1002/cam4.2754

**Published:** 2019-12-18

**Authors:** Guanzhang Li, You Zhai, Zheng Wang, Zhiliang Wang, Ruoyu Huang, Haoyu Jiang, Renpeng Li, Yuemei Feng, Yuanhao Chang, Tao Jiang, Wei Zhang

**Affiliations:** ^1^ Department of Molecular Neuropathology Beijing Neurosurgical Institute Capital Medical University Beijing China; ^2^ Department of Neurosurgery Beijing Tiantan Hospital Capital Medical University Beijing China; ^3^ Center of Brain Tumor Beijing Institute for Brain Disorders Beijing China; ^4^ China National Clinical Research Center for Neurological Diseases Beijing China; ^5^ Chinese Glioma Genome Atlas Network (CGGA) and Asian Glioma Genome Atlas Network (AGGA) Beijing China

**Keywords:** adjuvant therapy, age, genomic alteration, glioblastoma, prognosis

## Abstract

**Background:**

Glioblastoma is the most malignant tumor of the central nervous system. Several prediction models have been produced to aid in prognosis assessment. Age, a primary decision factor for prognosis, is associated with increased genomic alterations, however the exact link between increased age and poor prognosis is unknown.

**Objective:**

In this study, we aimed to reveal the underlying cause of poor prognosis in elderly patients.

**Methods:**

This study included data on 616 primary GBM tumor samples from the CGGA and TCGA databases and 41 nontumor brain tissue samples obtained from http://www.ncbi.nlm.nih.gov/geo/query/acc.cgi?acc=GSE53890. Hallmarks and clinicopathological characteristics were evaluated in both tumor and nontumor brain tissues. The association between choice of treatment regimen and age was measured using ANOVA and Student's *t* test.

**Results:**

Age was a robust predictor of poor prognosis in glioma. No age‐related hallmarks of cancer were detected, including pathological characteristics or mutations. However, treatment choice was strongly significantly different between old and young patients. Combined chemo‐radiation therapy could benefit old and young GBM patients, however, old patients are currently less likely to choose it.

**Conclusion:**

The vast divergence in prognosis between young and old GBM patients is largely caused by choice of treatment rather than age‐related tumor genomic characteristics. Postoperative standard radio‐ and chemotherapy provide strong benefits to primary glioblastoma patients of all ages.

## Graphical abstract

The main reason of poorer prognosis in elder patients was conservative treatment choosing, while standard radio‐ and chemotherapy should be generalized in all age grade of patients.

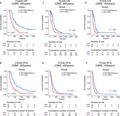

## 1 INTRODUCTION

Glioma is the most common and lethal type of intracranial malignancy.^1^ In clinical practice, even after aggressive treatment, the prognosis of glioma patients is still poor. The median survival of glioblastoma (GBM) is about 14.4 months.^2^, ^3^ Numerous prediction models based on clinical and clinicopathological characteristics have been developed to aid in individual diagnosis and to guide treatment.^4^, ^5^, ^6^, ^7^

For the last few years, several clinicopathological characteristics have shown a promising correlation with treatment and prognosis, including EGFR amplification,^8^ IDH mutation, MGMT promoter methylation status^9^, ^10^ and GBM transcriptome subtypes.^11^, ^12^ Among all characteristics, patient age seems to be one of the most definitive prognostic factors.^13^ In clinical practice, we have also found that older patients have worse outcomes than younger ones. This observation led us to consider whether the worse overall survival is due to antineoplastic protocols or is instead caused by some sort of age‐dependent gene expression in GBM. With the help of the cancer genome atlas (TCGA), we were able to screen out several prognostic biomarkers with greater accuracy. Subdivision of patient groups provided explanation for some different properties of GBMs in a different hierarchy. However, the details of the treatment that patients received has not received much attention, and that might be a core explanatory factor for the prognosis of older patients.

In this article, we aimed to reveal the relationship between age and GBM prognosis. We assumed that there exist several age‐associated genomic changes that may influence the prognosis of elder patients. First, we verified the relationship between age and prognosis in primary GBMs. The relationship between specific biofunctional characteristics of the tumor and age was analyzed. Furthermore, the correlations between known molecular biomarkers of GBM and age were also calculated. Unexpectedly, no age‐related biofunction, pathological, or molecular characteristics were detected. Intriguingly, in our follow‐up studies, we found that older patients tended to choose conservative treatment while younger patients underwent radio‐ and chemotherapy. It was these differences in treatment that led to the poorer prognoses in older GBM patients. Since no significant genomic changes contribute to poorer outcomes for elder patients, the choice of treatment seems to be a core issue determining prognosis. Hence, we propose a standardized treatment, including surgical medical care and radio‐ plus chemotherapy, for all patients, regardless of age.

## 2 METHODS

### 2.1 Patients and clinical information

This study included a total of 616 primary GBM patients from the CGGA (http://www.cgga.org.cn) and TCGA ((http://cancergenome.nih.gov) databases and 41 nontumor patients from http://www.ncbi.nlm.nih.gov/geo/query/acc.cgi?acc=GSE53890 (https://www.ncbi.nlm.nih.gov/geo/query/acc.cgi?acc=GSE53890). All tumor and nontumor brain samples with transcriptomic microarray data were used for biological profiling. Information on the above patients is available from the corresponding data portal. The characteristics of all patients are summarized in Table 1.

**Table 1:** Clinical information of patients

Characteristics (CGGA)	No. of patients (CGGA)
Age at diagnosis	
≤65	103
>65	6
Preoperative KPS score	
≥80	31
<80	26
TCGA subtypes	
Proneural	16
Neural	8
Classical	15
Mesenchymal	70
Radiotherapy + TMZ chemotherapy	
Yes	50
No	59
Radiotherapy	
Yes	78
No	31
TMZ chemotherapy	
Yes	50
No	59
IDH1/2 mutation	
Mutation	13
Wildtype	96
MGMG promotor status	
Methylation	40
Unmethylation	62
TP53 mutation	
Mutation	14
Wildtype	61
EGFR amplification	
Amplification	6
Wildtype	88

Characteristics (TCGA)	No. of Patients (TCGA)
Age at diagnosis	
≤65	336
>65	184
Preoperative KPS score	
≥80	274
<80	101
TCGA subtypes	
Proneural	98
Neural	53
Classical	145
Mesenchymal	172
Radiotherapy + TMZ chemotherapy	
Yes	335
No	155
Radiotherapy	
Yes	479
No	11
TMZ chemotherapy	
Yes	346
No	144
IDH1/2 mutation	
Mutation	24
Wildtype	367
MGMG status	
Methylation	151
Unmethylation	184
TP53 mutation	
Mutation	64
Wildtype	191
EGFR amplification	
Amplification	52
Wildtype	203

Note

Number of primary GBM patients engaged in our study was listed. All patients were stratified with age, clinicopathological characteristics, and treatment options respectively. Number of patients engaged in each step of analysis was listed.

### 2.2 The hallmark scores

The hallmark scores were calculated by Gene Set Variation Analysis (GSVA) analysis. GSVA analysis was performed using the *gsva* package in software environment R (version 3.5.0). The hallmarks gene list was downloaded from the GSEA Web portal (http://software.broadinstitute.org/gsea/msigdb/index.jsp). The analysis process was performed using the default parameters. After *pearson* correlation analysis, the age‐related hallmark was displayed by a scatter diagram.

### 2.3 Statistical analysis

Statistical analyses were performed using R 3.5.0, SPSS software 25.0 and GraphPad Prism 7.0. A Kaplan‐Meier survival curve was built to estimate the survival distributions based on a corresponding median value. The log‐rank test was used to assess the statistical significance of differences between survival groups. Differences in continuous variables between groups were evaluated by the Student's *t* test and four groups were evaluated by one‐way ANOVA. Correlation between two variables was analyzed by *pearson* correlation analysis. Patients with missing information were excluded from the corresponding analysis. A *P *< .05 was regarded as statistically significant.

## 3 RESULTS

### 3.1 Older patients have a worse prognosis

**Figure 1:**
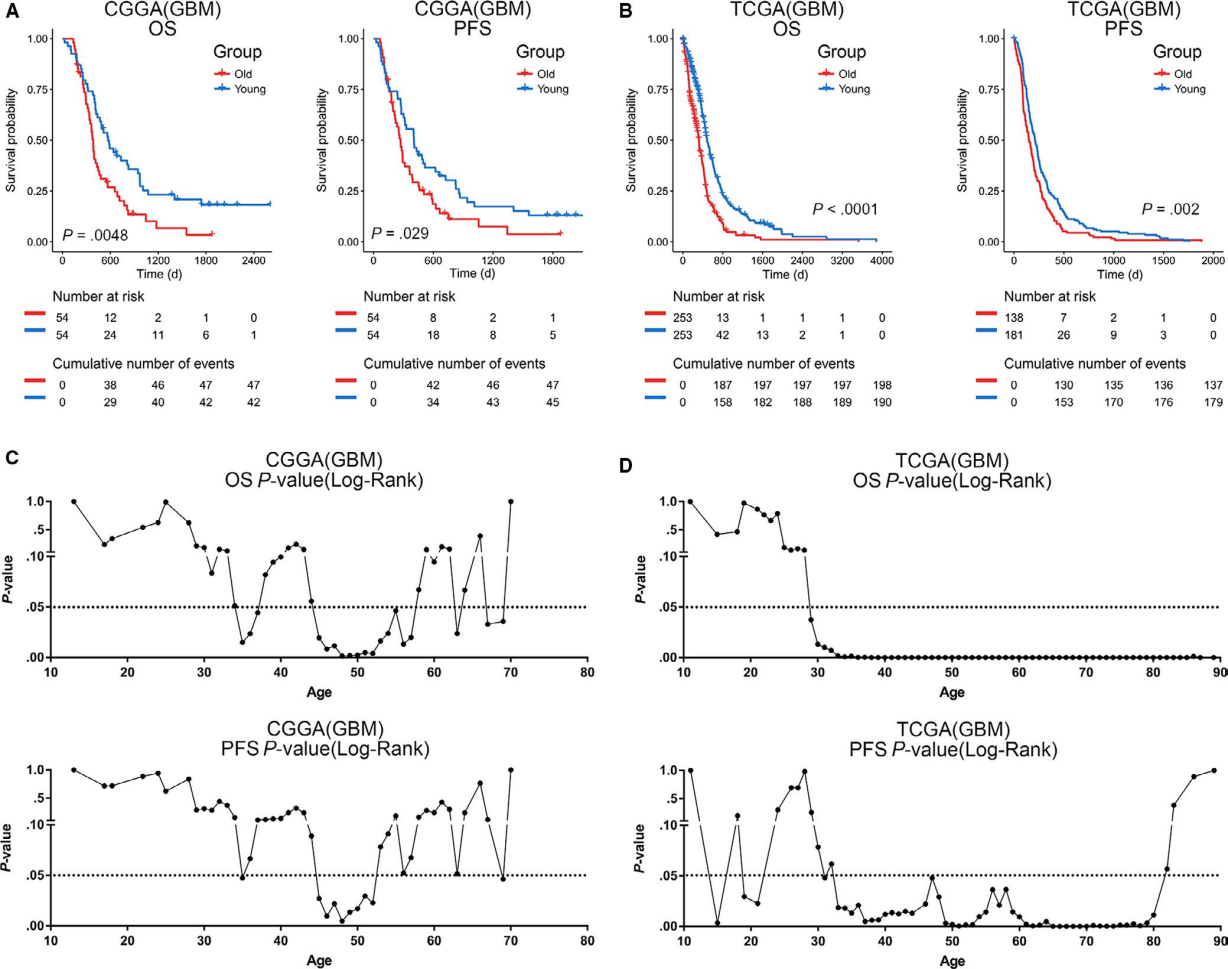
Survival difference between old and young GBM patients. (A and B) Statistical difference in survival between the old and young group. Older patients have a poorer OS and PFS prognosis. (C and D) *P*‐value between prognosis of patients using arbitrary ages as the cut‐off. Points below the black dotted line represented statistically significant differences in prognosis. The number at risk and the cumulative number of events are important parameters of Kaplan‐Meier survival analysis. The number at risk represents the number of subjects at risk at the corresponding time. Cumulative number of events represents the total number of deaths at the corresponding time. The log‐rank test was used to assess the statistical significance of stratified survival groups

First, the Kaplan‐Meier curve showed a distinct difference between old and young patients with primary GBM in terms of overall survival and progression‐free survival (Figure 1A,B). To further validate the relationship between age and prognosis in GBM patients, a Kaplan‐Meier survival analysis using continuous cutoff values was performed. As shown in Figure 1C,D, the prognosis analysis for all groups except those over 60 years showed a stable prognostic significance. Importantly, the above analysis can be verified in two independent databases, the CGGA and TCGA Databases.

### 3.2 Tumor biofunction and genomic characteristics did not correlate with age

**Figure 2:**
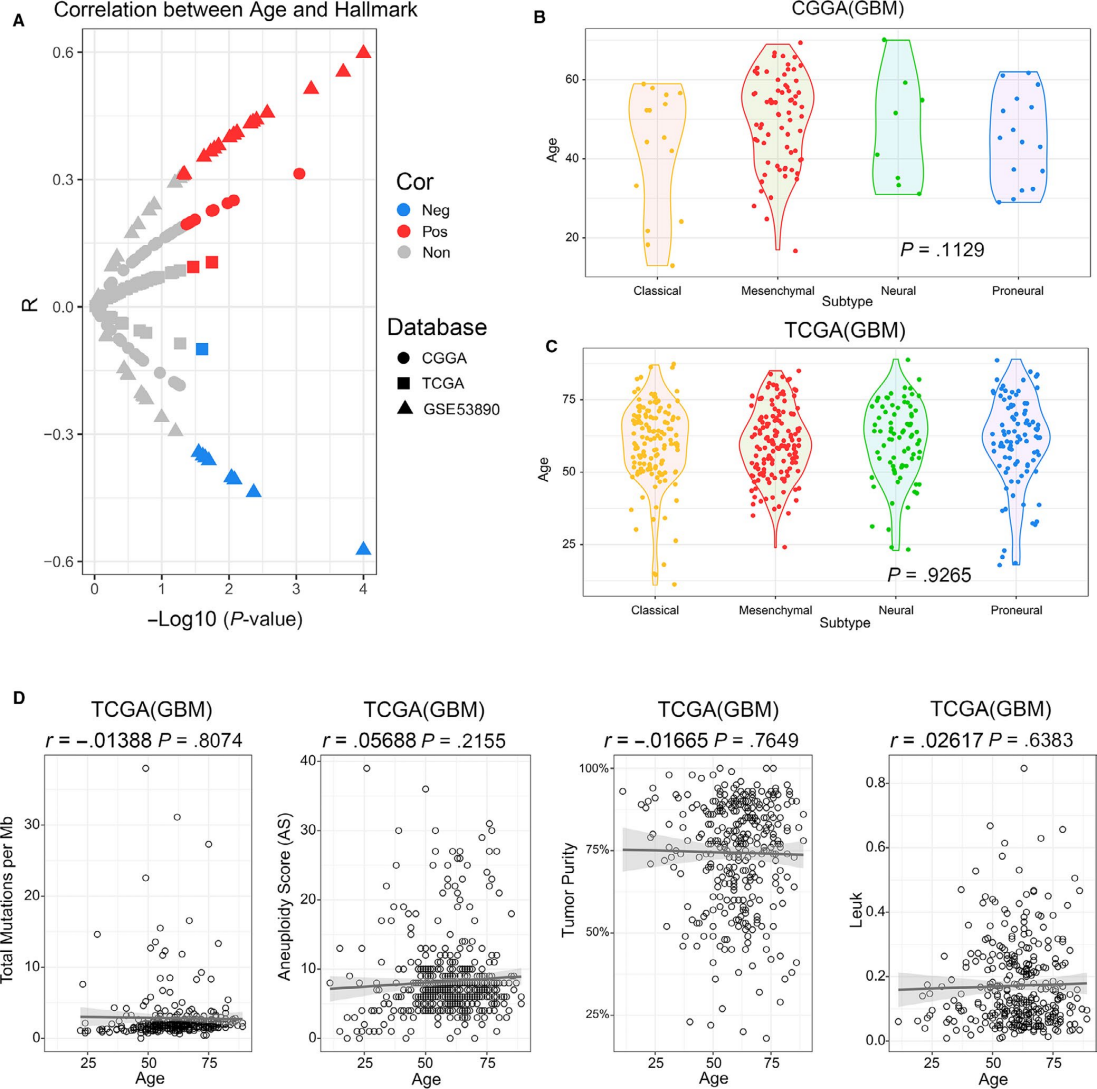
Correlation between biofunction, genomic characteristics, and age. A, Age distribution of hallmarks of cancer in normal and GBM samples. Both normal and tumor tissue have hallmarks positively or negatively correlated with age. *R* and *P* values were obtained from the pearson correlation analysis between age and hallmark scores. A significant positive correlation was represented by red dots. A significant negative correlation was represented by blue dots. Gray dots represented no significant correlation. The calculation method of the hallmark scores is detailed in the methods section. B and C, Age distribution of GBM transcriptome subtypes. The yellow dots represent the classical subtype. The red dots represent the mesenchymal subtype. The green dots represent the neural subtype. The blue dots represent the proneural subtype. No significant difference was observed among the 4 subtypes. One‐way ANOVA analysis was performed to assess the statistical significance of variance. D, Overall genomic characteristics distribution ranked by age. No statistical difference was observed as age increased. Pearson correlation analysis was performed to assess the statistical significance between age and genome characteristics

In order to explore the factors that cause poor prognosis in high‐age patients, we analyzed the relationship between age and genetic alterations in primary GBM. The hallmarks of cancer are widely recognized features of tumor biological behavior.^14^, ^15^ Therefore, we next identified the correlation between age and cancer hallmarks. Unexpectedly, though several hallmarks of tumor‐related biofunction were significantly associated with age, GBM samples did not show more cancer‐specific features than normal brain samples (Figure 2A). This result implied that cancer hallmarks correlated with age in primary GBM were not independent prognostic factors, since they are much less correlated with age than normal tissue. Furthermore, no statistical difference was observed in GBM transcriptome subtypes among the different age groups (Figure 2B,C). Next, we split tumor pathological characteristics into 4 aspects, including total mutations, aneuploidy, purity, and leukocyte infiltration. As shown in Figure 2D, no significant change was observed in these 4 aspects with increasing age. Together, these results suggested that tumor samples from different ages shared a common biological characteristic at the overall genome level. Therefore, continued efforts to identify other factors that might explain the relationship between age and prognosis were required.

### 3.3 Known molecular pathology mutations did not cause worse prognosis for older patients

**Figure 3:**
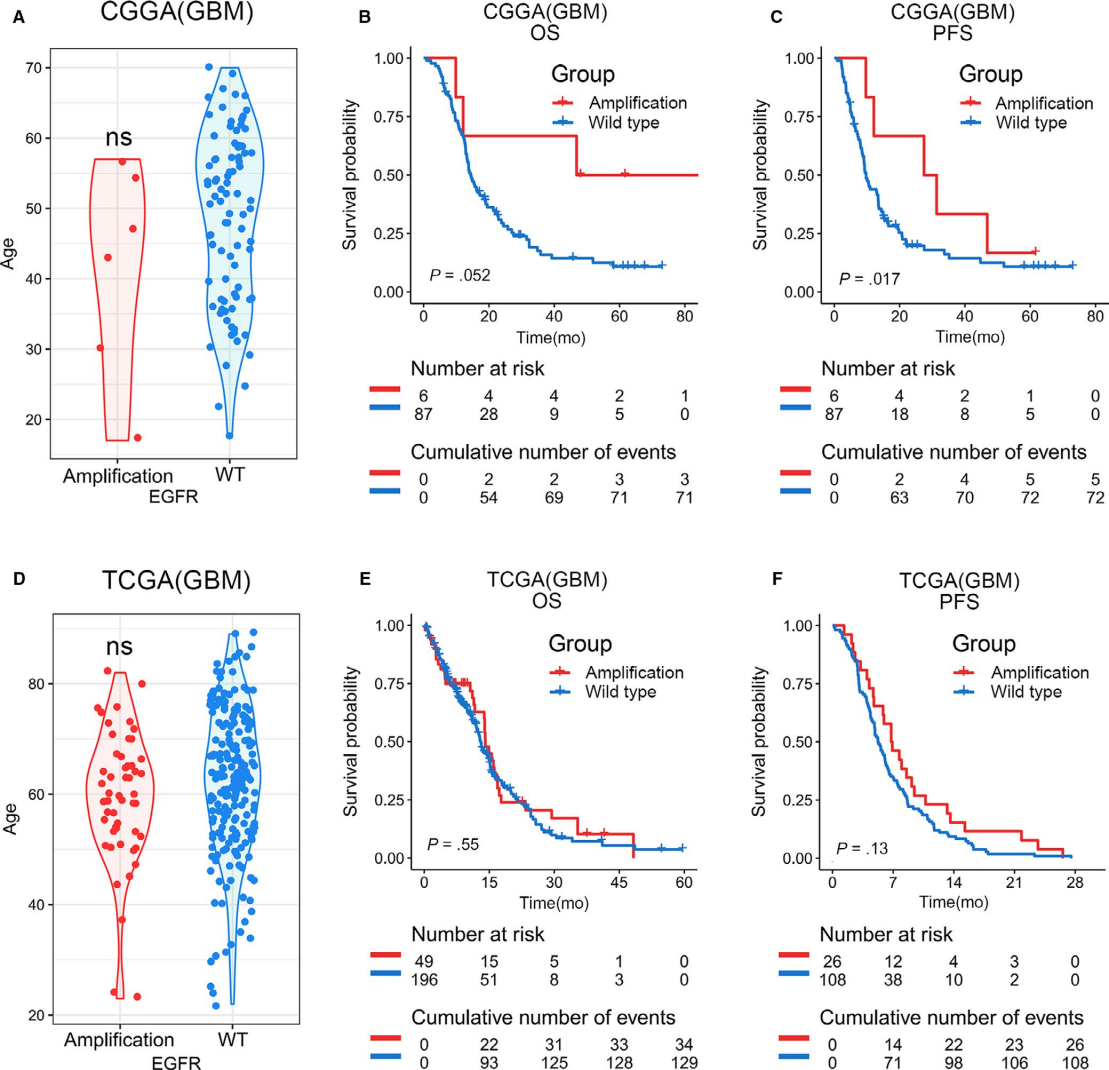
Age distribution in EGFR amplification status in primary GBM. A and D, No significant difference was observed across age groups between amplification and wildtype EGFR status in CGGA and TCGA databases. An unpaired t test was used in the differential analysis. B, C, E and F, Kaplan‐Meier survival analysis for EGFR amplification status in GBM. The log‐rank test was used in survival analysis

Epidermal Growth Factor Receptor (EGFR) amplification is one of the most common genomic alterations in GBM (Figure 3). As shown in Figure 3A,D, no age‐related significant differences were detected between the distribution of amplification type and wild type in either the TCGA or CGGA database. Patients with different states of EGFR appear to show different overall survival and progression‐free survival in CGGA database (Figure 3B,C). However, in the TCGA database, there was no significant correlation between age and EGFR amplification status in GBM patients (Figure 3E,F). Taken together, the reason for the poor prognosis of elderly GBM patients was not likely to be related to increased prevalence of EGFR amplification.

**Figure 4:**
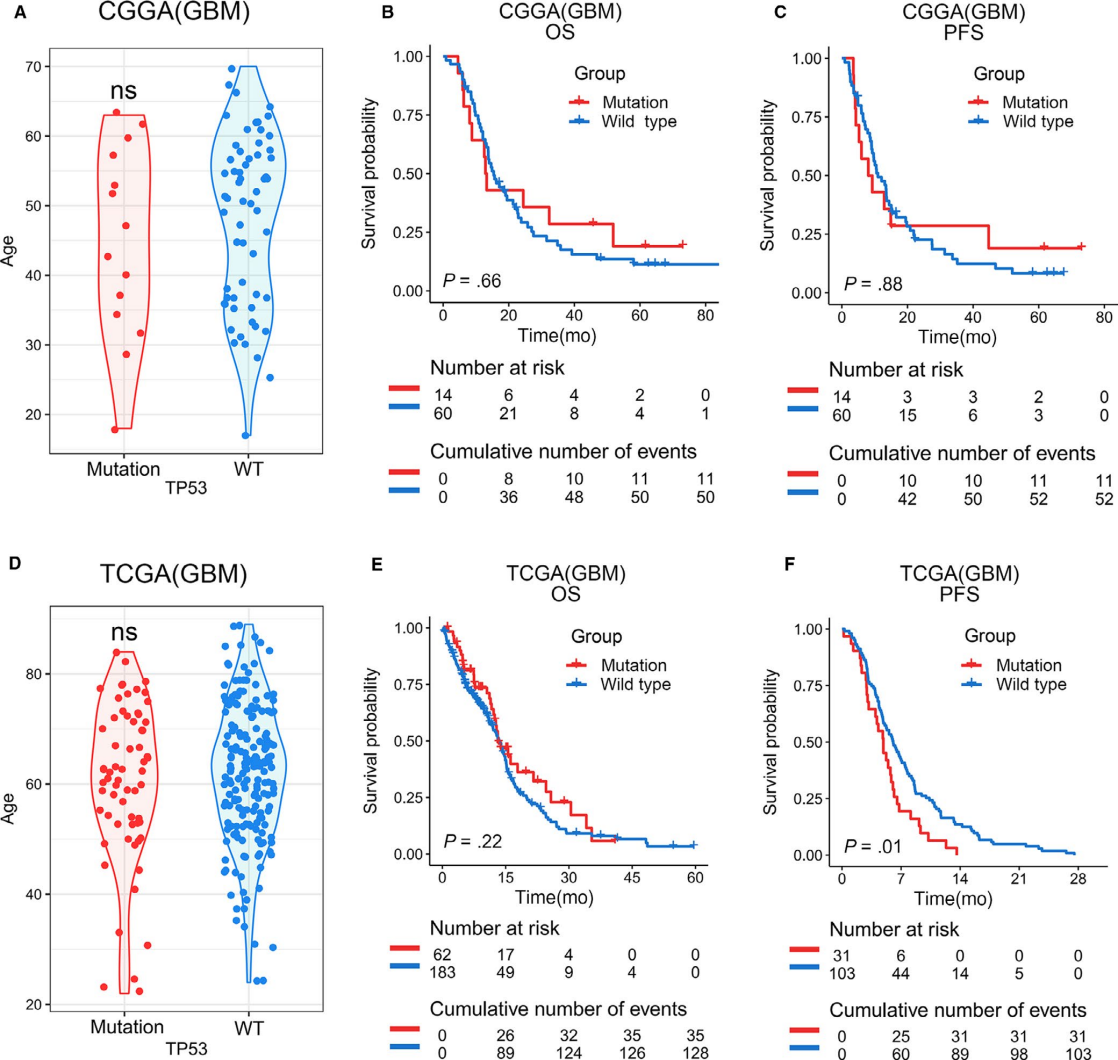
Age distribution in TP53 mutation status in primary GBM. A and D, No significant difference across age groups was observed between TP53 mutation and wild‐type GBMs. An unpaired t test was used in the differential analysis. B, C, E and F, Kaplan‐Meier survival analysis of TP53 mutation status. Statistically different PFS was observed in the TCGA database between TP53 mutation and wild‐type group (*P* = .01). The log‐rank test was used in survival analysis

We further analyzed whether TP53 mutation status, another high‐frequency mutation in GBM, was correlated with age and survival (Figure 4). Similar to our findings with EGFR, no statistical difference was observed in the distribution of age between mutation group and wild‐type group (Figure 4A,D). Furthermore, no distinct differences in OS or PFS survival were associated with TP53 mutation status in either the CGGA or TCGA database (Figure 4B‐F). These results indicated that there was no correlation between TP53 mutation status and either age or prognosis of patients with GBM.

Finally, MGMT promoter methylation status was analyzed for an association with either age or prognosis. In good agreement with our prior findings, age was not associated with MGMT status. Indeed, both methylated status and unmethylated status are evenly distributed with age, indicating that there was no correlation between age and MGMT status (Figure S1A,D).

To sum up these findings, known molecular pathology mutations did not appear to cause poorer prognosis in primary GBMs. In addition, the age of onset was not correlated with these factors. These results contradict the assumption that worse survival in elderly patients is caused by age‐related genomic or transcriptomic alterations in glioma.

### 3.4 Treatment options were divergent in young and old patients and were associated with worse outcomes in old patients

**Figure 5:**
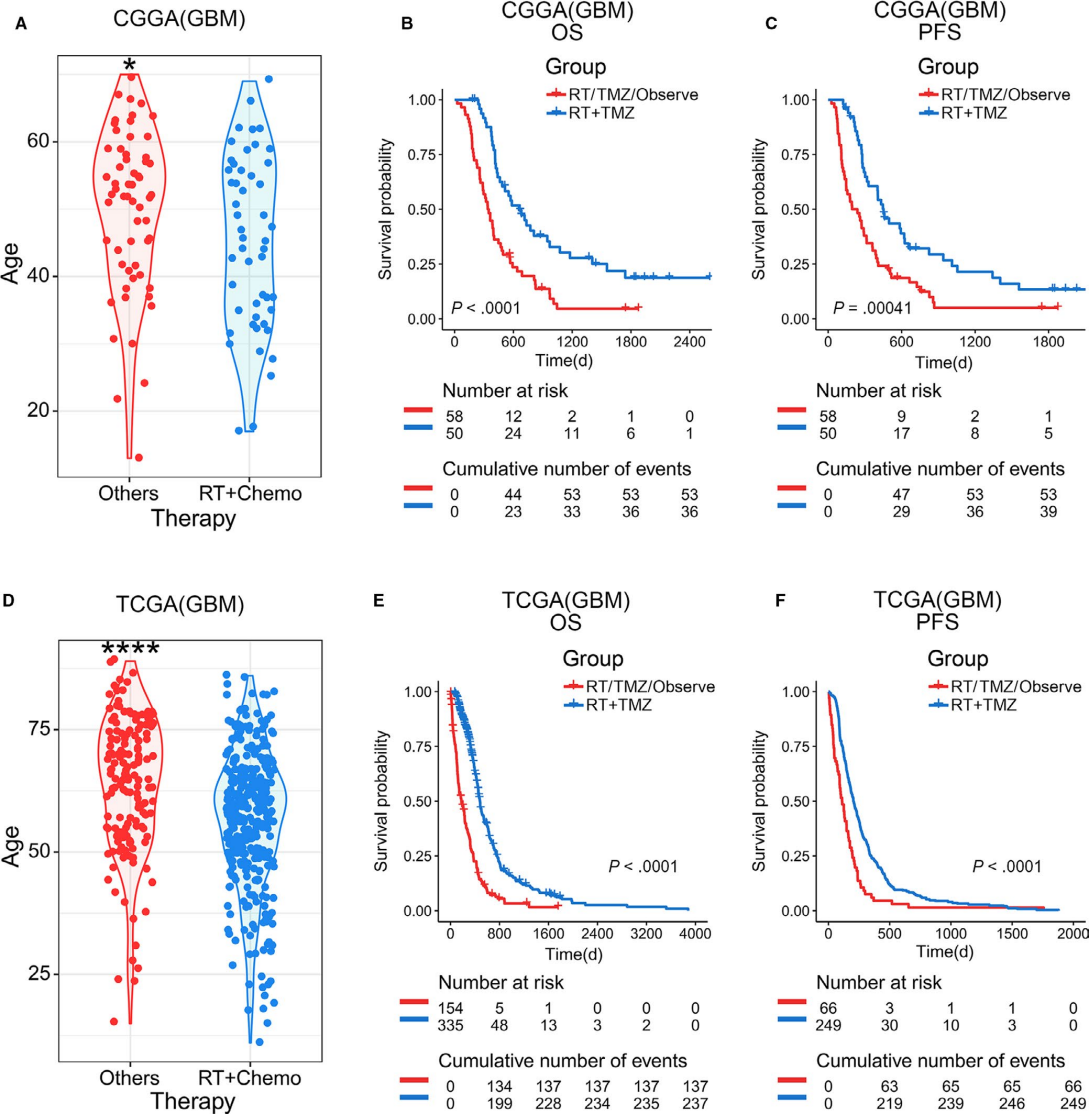
Treatment options in young and elder GBM patients. A and D, Age distribution of different treatment options. Younger patients tended to receive standardized combination therapy more often in CGGA and TCGA databases. The unpaired *t* test was used in the differential analysis. B, C, E, and F, Kaplan‐Meier survival analysis for different treatment options. Patients who received standardized postoperative adjuvant therapy had significantly longer overall and PFS survival time. RT + TMZ represents patients undergoing postoperative adjuvant radiotherapy and TMZ chemotherapy combined therapy. RT/TMZ/Observe means patients only receive RT or TMZ or Observe after surgery. The log‐rank test was used in survival analysis

Differences in treatment preferences are common in clinical practice but are often ignored in prognosis analysis. Both Asian and North American GBM patients had different treatment preferences as a function of age. In general, older patients tended to choose conservative treatment, and younger patients chose standardized radio‐ and chemotherapy after surgical care (Figure 5A,D). However, clinicians have ignored such a fact that standardized postoperative adjuvant therapy ensured promising prognosis for patients compared with any other therapies in primary GBMs (Figure 5B‐F). Therefore, further analysis was performed to verify whether the poor prognosis of elderly GBM patients was caused by therapeutic preference.

### 3.5 Postoperative standard adjuvant therapy can significantly benefit both young and old gbm patients

**Figure 6:**
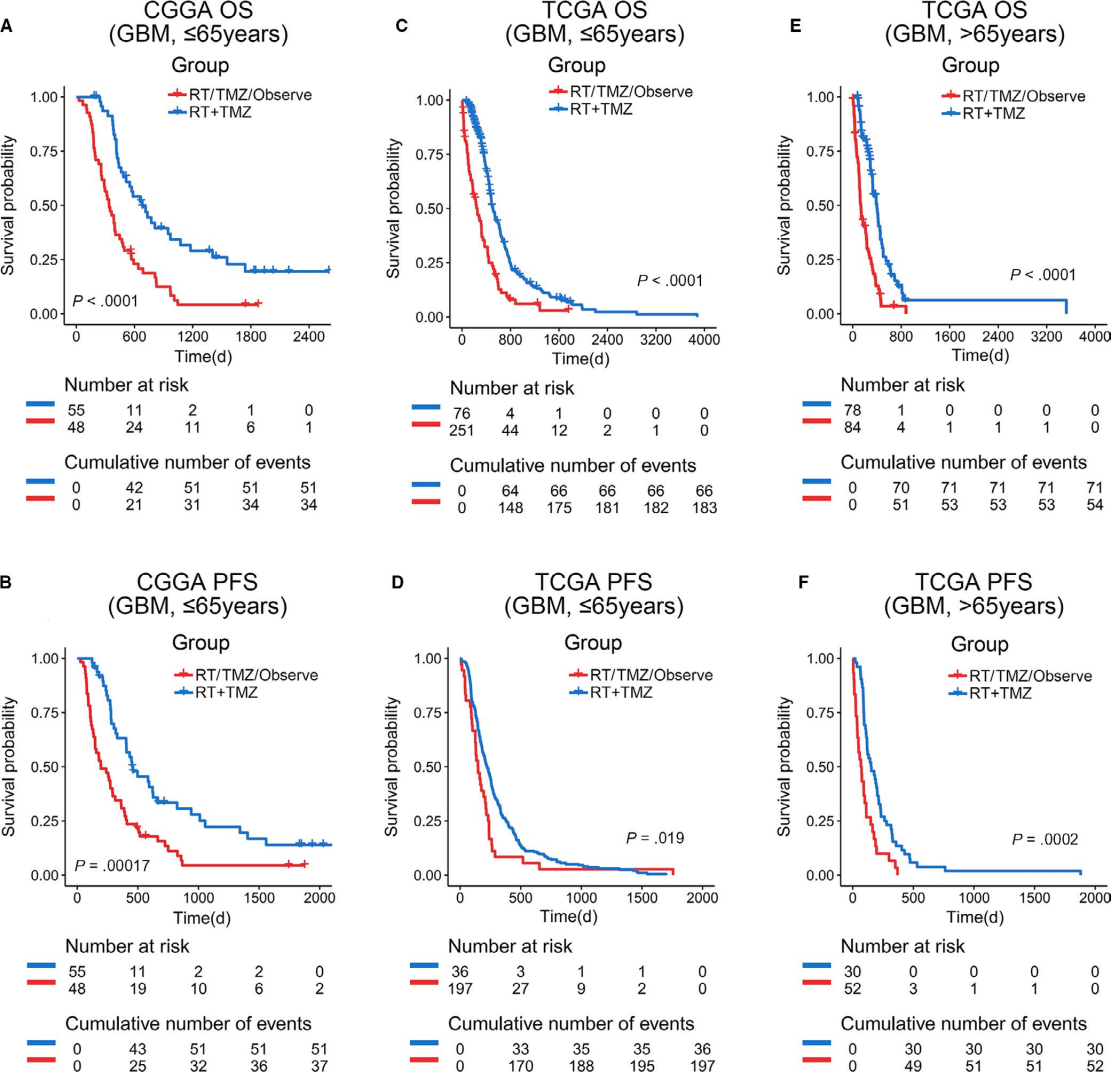
Postoperative standard adjuvant therapy benefited all age grades of GBM patients. A and B, Younger patients who received combination therapy had a better prognosis than those did not (*P* < .0001 and *P* = .00017 in OS and PFS survival analysis respectively). C and D, Results were verified by the TCGA database (OS, *P* < .0001 and PFS, *P* = .019). E and F, Older patients could also benefit from combination therapy (OS, *P* < .0001 and PFS, *P* = .0002)

Patients were divided into older (age ≤ 65) and younger (age > 65) groups. Given that there were only 6 patients older than 65 years in CGGA, we did not perform this analysis on that data set. As illustrated in Figure 6A‐D, patients younger than 65 years significantly benefited from radio and temozolomide (TMZ) combination therapy. On the contrary, another other choice of treatment was associated with a relatively worse prognosis in both the TCGA and CGGA databases. Importantly, standard combination therapy also markedly prolonged the survival of patients above 65 in TCGA (Figure 6E,F), compared with other adjuvant treatments. These results highlighted the essential inclusion of postoperative standard adjuvant therapy for all primary GBM patients, regardless of their age.

## 4 DISCUSSION

With the continuous advancement of genome sequencing technology, our understanding of cancer is becoming increasingly comprehensive. It is commonly suggested that genomic alterations trigger tumorigenesis and lead to poor prognoses.^16^, ^17^, ^18^ At the same time, other traditional clinical features are gradually being left out from prognostic consideration. However, it is well‐documented that age is a stable prognostic factor for primary GBM.^19^ Thus, it has been assumed that the reason for worse prognoses in older patients is due to increased genomic mutations in the tumors of older patients. To verify the validity of this hypothesis, we conducted this study.

Given that age is certainly suggested to be a risk factor for prognosis, we compared the prognosis for different age groups. As a whole, older patients had statistically significantly poorer outcomes. In order to verify whether the tumors of elderly patients have more genomic alterations that lead to poor prognosis, several factors were investigated for their correlation with age. The “Hallmarks of Cancer” was used to categorize characteristic biological functions of the tumor, as previously described.^15^ Correlation analysis found some hallmark changes were associated with age of GBM patients. However, the extent of mutations in tumor samples from the CGGA or TCGA databases were much less than in non‐tumor samples. Therefore, age‐related hallmarks in tumor samples cannot be considered to be specific age‐associated risk factors. Similarly, the GBM transcriptome subtype, another important genomic feature in GBM,^20^ showed no significant age‐related divergence. Furthermore, other genomic features, including total mutations counts per Mb, aneuploidy, tumor purity, and lymphocyte proportion in the tumor also showed no statistical relationship with patient age. EGFR amplification and TP53 mutation are high frequency genomic alterations in primary GBM.^21^, ^22^, ^23^ To date, there have been few studies focused on the relationship between these mutations and age in primary GBM. There was no significant correlation between EGFR or TP53 and age in our study. These unexpected results reject the hypothesis that age‐associated clinicopathologic characteristics might trigger poorer prognosis. In turn, we focused on the choice treatment options among patients of different ages.

Surprisingly, there was a statistically significant correlation between age and choice of treatment. Most patients undergoing standard chemoradiotherapy after surgery were younger patients. On the contrary, older patients typically chose more conservative treatment. This phenomenon was common in GBM clinical treatment in both China and the United States. Although it has been clear that postoperative standard adjuvant therapy benefits primary GBM patients in general, it was not known whether the standard treatment was equally beneficial among elderly patients. Therefore, some doctors considered the physical condition of elderly patients and preferred to choose a conservative treatment. This study found that the biologic properties of GBM are not significantly different among patients of different ages. Instead, the relatively poor prognosis of older patients is primarily caused by the selection of conservative treatment options.

## 5 CONCLUSION

After analyzing several known clinicopathology characteristics and finding no difference across age groups, we focused on the clinical treatment method patients underwent. Here, we found that choice of conservative therapy was associated with a significantly worse prognosis relative to standard chemoradiotherapy after surgery. According to the results of this study, we suggest that all GBM patients should receive postoperative standard radio‐ and chemotherapy. In short, postoperative standard radio‐ and chemotherapy appears to benefit all ages of primary GBM patients.

## CONFLICT OF INTEREST

The authors declare no potential conflicts of interest.

## ETHICS APPROVAL AND CONSENT TO PARTICIPATE

This study was approved by Beijing Tiantan Hospital institutional review board (IRB). All patients provided written informed consent for the publication of all associated data in this study.

## Data Availability

The datasets used and/or analyzed during the current study are available from the corresponding author on reasonable request.
