# Supplementary figures and images for: Postoperative standard chemoradiotherapy benefits primary glioblastoma patients of all ages
Source: Cancer Med. 2019 Dec 18;9(6):1955–65. doi: 10.1002/cam4.2754 (PMC7064041; doi:10.1002/cam4.2754)

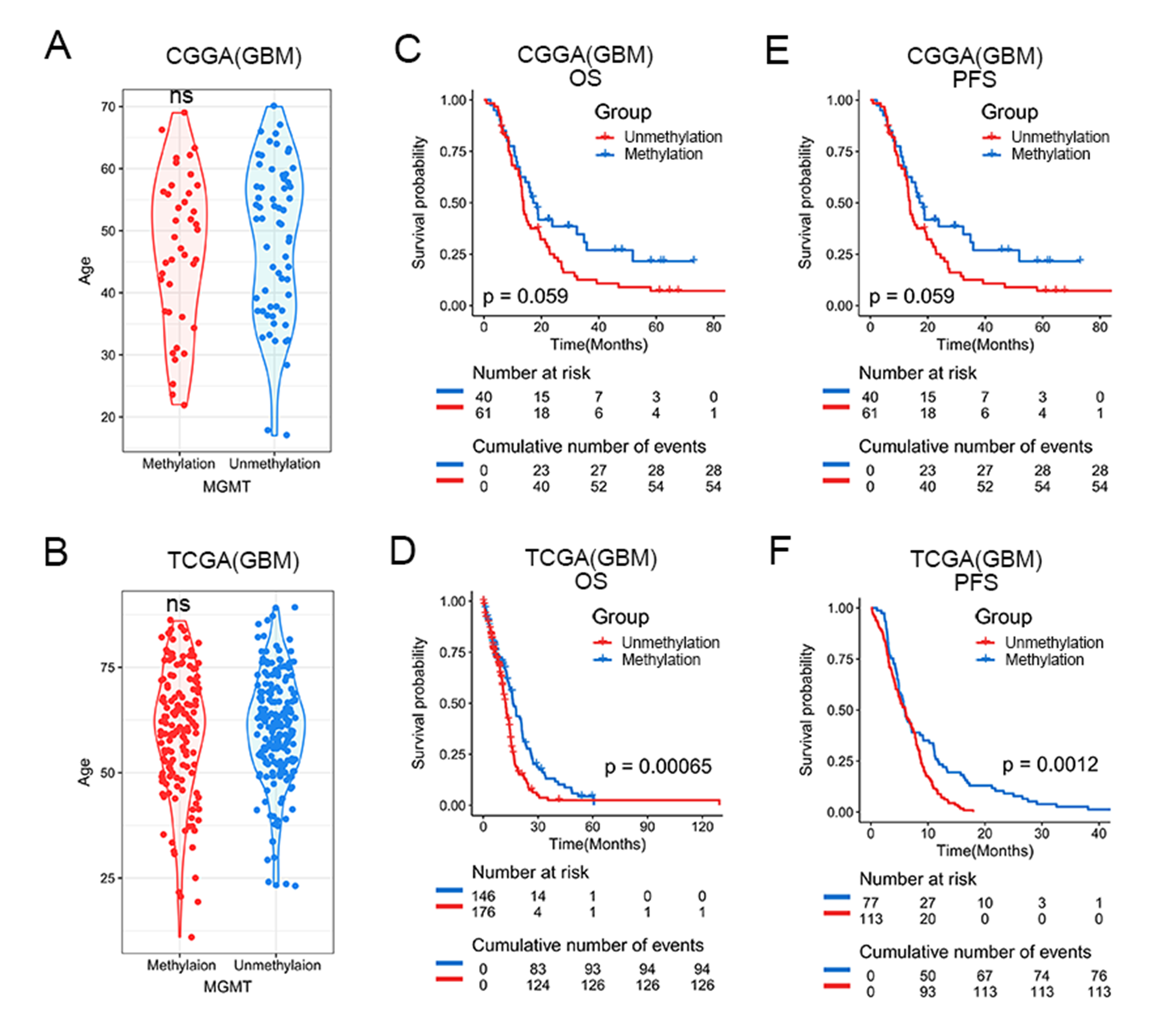

Supplement: Supplementary file 1 [file CAM4-9-1955-s001.tif]

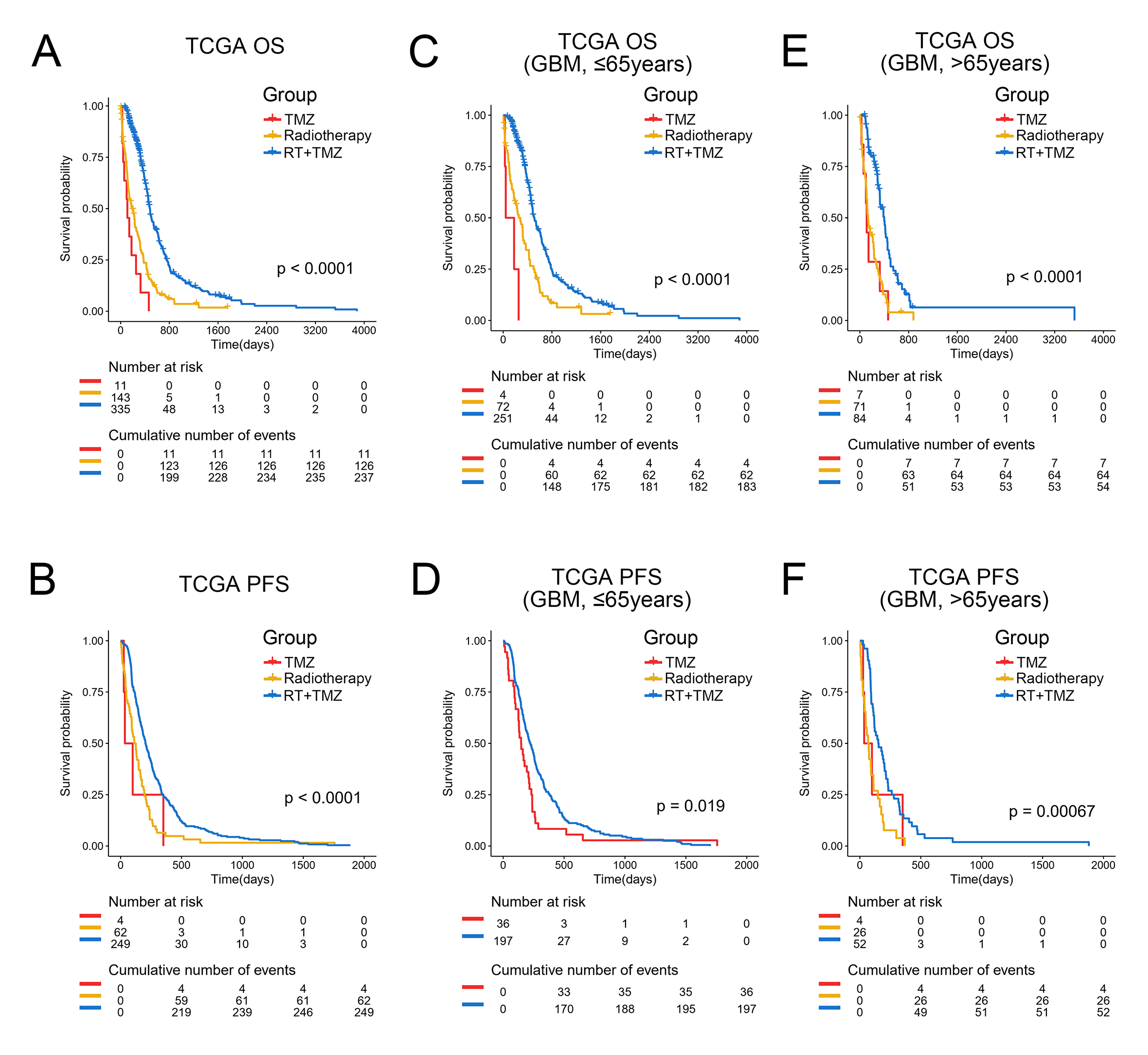

Supplement: Supplementary file 2 [file CAM4-9-1955-s002.tif]

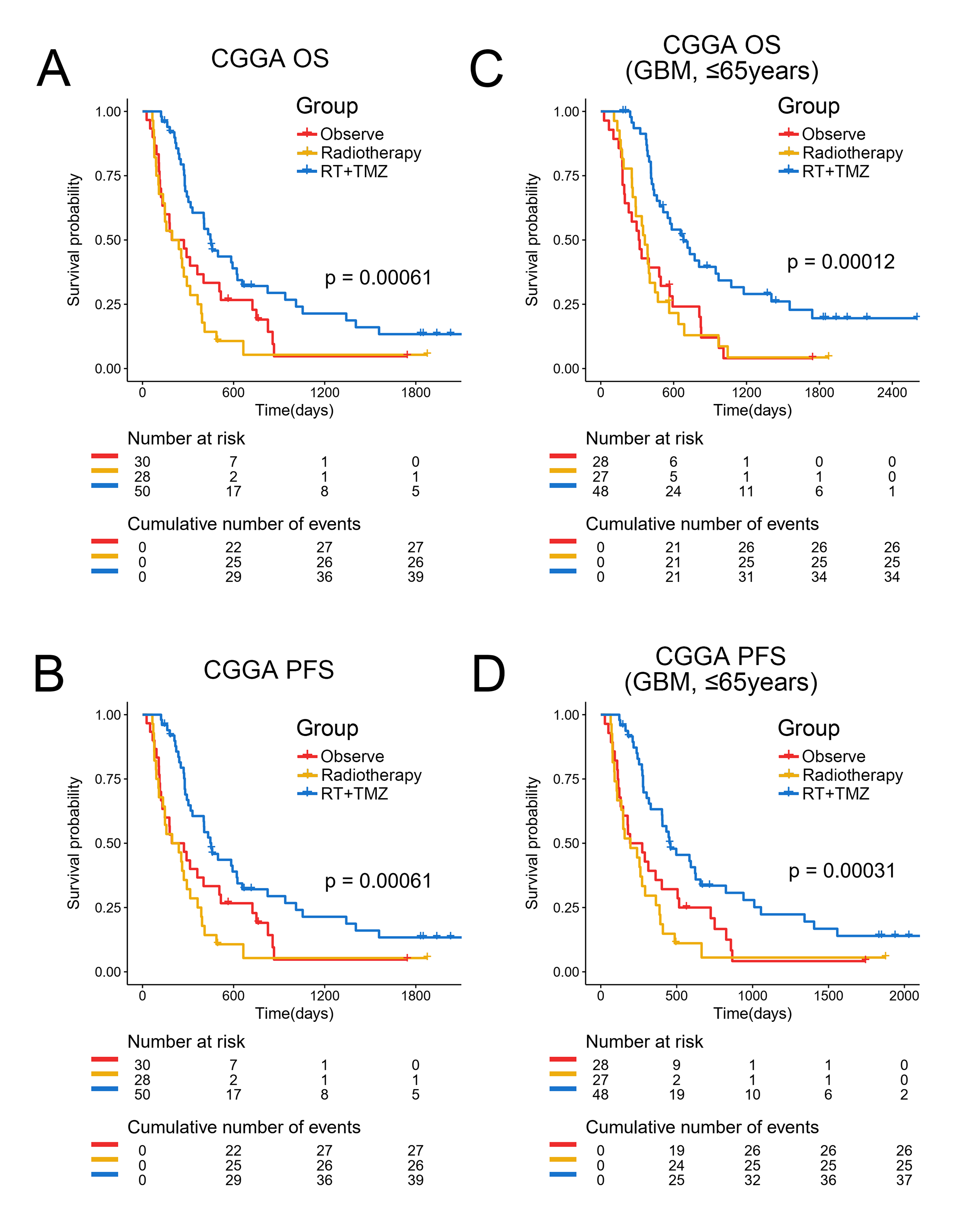

Supplement: Supplementary file 3 [file CAM4-9-1955-s003.tif]
